# Diagnostic tests for progestogen hypersensitivity

**DOI:** 10.3389/falgy.2024.1384140

**Published:** 2024-04-24

**Authors:** César Daniel Alonso Bello, Otto Pavel González Guzmán, Carol Vivian Moncayo Coello, María Isabel Rojo Gutiérrez, María Isabel Castrejón Vázquez

**Affiliations:** ^1^ Allergy and Immunology Service, Hospital Juárez de México, Secretaría de Salud, Mexico City, Mexico; ^2^ Spine Rehabilitation Service and Osteoporosis Clinic, Instituto Nacional de Rehabilitación, Mexico City, Mexico; ^3^ Allergy and Immunology Service, Medical Director, Unidad Médica Zurich Satélite, Naucalpan, Mexico; ^4^ Clinical Immunology and Allergy Service, Centro Médico Nacional 20 de Noviembre, Instituto de Seguridad y Servicios Sociales de los Trabajadores del Estado, Mexico City, Mexico

**Keywords:** progesterone, autoimmune diseases, menstrual cycle, urticaria, allergy

## Abstract

Progesterone is an endogenous hormone, produced by the adrenal cortex, the gonads and in women, its source is the corpus luteum. Progesterone is produced in the late phase of the menstrual cycle, when implantation of the zygote does not occur, the corpus luteum involutes and the release of progesterone is suppressed, thus initiating menstruation. Progestogen Hypersensitivity were initially identified as hormone allergy and were related to endogenous reactions to hormones and alteration of ovarian function. Skin manifestations such as dermatitis or urticaria were initially reported and described as progesterone autoimmune dermatitis, although the immune-mediated mechanism was not clear. Currently there is no standardization for *in vivo* or *in vitro* tests for Progestogen Hypersensitivity diagnosis. In this review, we will address the different diagnostic methods of this disease.

## 1 Introduction

Progestogen Hypersensitivity (PH) were initially identified as hormone allergy and were related to endogenous reactions to hormones and alteration of ovarian function (1). In 1947, Zondek and Bromnberg described hypersensitivity mechanisms related to endogenous hormones; These reactions were demonstrated by intradermal application tests of insulin and gonadotropin (2).

William Finch, in his article “The etiology of nausea and vomiting of pregnancy”, published in 1938, postulated that nausea during pregnancy was related to the functionality of the corpus luteum. The skin test performed to demonstrate sensitization and the desensitization scheme proposed in the case series of 30 patients were not standardized; the patients did not present skin symptoms or clinical manifestations additional to nausea; however, they presented erythema reactions at the site of intradermal application of progestin (3). This case series served as a background to recognize hypersensitivity reactions to endogenous progesterone.

The concept of allergy has evolved; today it is defined as an abnormal, unexpected, or exaggerated reaction to an external stimulus that involves the immune system. However, although the definition may be ambiguous, the current and recently published classification of the allergy European Academy of Allergy and Clinical Immunology (EAACI) helps us understand the mechanisms of how allergic pathologies are mediated by different immunological mechanisms; Thus, the classification of allergic mechanisms is classified into four main groups: antibody-mediated reactions, cell-mediated reactions, Tissue-driven mechanism and direct response to chemicals. In this way, the panorama opens up more, and the immunological mechanisms that can be considered allergy are recognized (4).

For this reason, we will call the reactions presented to endogenous or exogenous progesterone that we will study below, Progestogen Hypersensitivity, in this way we include any immunological mechanism that is currently classified as an allergy.

## 2 Progesterone in immunoendrocrinology

Progesterone (P4) was one of the first hormones identified, it is known for its role as a sex steroid (5). P4 is an endogenous 21-carbon steroid hormone synthesized from cholesterol in the corpus luteum of the ovaries and also by the placenta during pregnancy. To a lesser extent, P4 is also produced in the adrenal cortex, Leydig cells, adipose tissue, and the nervous system, being synthesized in both neurons and glia (6, 7).

Progesterone concentrations vary throughout the life course, presenting their greatest fluctuations during puberty and dynamic changes during the menstrual cycle in response to the pulsatile secretion of FSH and LH by the pituitary gland. Its levels are considerably higher in women than in men. During pregnancy, P4 increases its levels constantly and remains elevated throughout pregnancy, being essential for pregnancy retention; after childbirth, there is a rapid decrease in its serum concentration (8).

The immunomodulatory properties of progesterone have been described for several years in experimental models (9). Its role in tolerance towards paternal antigens during pregnancy is the best-known model. The presence of P4 nuclear receptors (PR) in the cells of the immune system has been strongly associated with pregnancy; most studies have shown the absence of PR in leukocytes in peripheral blood in non-pregnant women (10), However, the immunomodulatory actions of progesterone can also be mediated by pathways independent of its nuclear receptor, pathways that seem to be relevant in the regulation of immunity in people outside of pregnancy (11). A high percentage of RP-positive lymphocytes has been detected. in the peripheral blood of patients with liver transplants or transfusions (12), suggesting that the activation of lymphocytes resulting from permanent alloantigenic stimulation could induce the expression of RP in lymphocytes, in the setting of pregnancy the continuous exposure of the mother to fetal antigens could be a mechanism of induction of these receptors.

P4 has nuclear and non-nuclear pathways, the complex formed by progesterone and its nuclear receptor (RPn) regulates cellular functions through direct nuclear genomic pathways that affect gene expression and transcription, indirect genomic pathways are mediated by linked receptors to the membrane and cell surface that activate and modulate second messengers and ion channels to exert their genomic effects (13).

The RPn have two main isoforms PR-A and PR-B, these bind to P4 and translocate to the nucleus to join with the response elements. Although both isoforms are transcribed by the same gene, these are functionally different receptors and the relationship PR-A:PR-B determines the impact of P4 on cellular transcriptional activity (14). In PRs found in various non-reproductive tissues, including lymphoid, intestine and brain, progesterone may promote a primarily inhibitory effect on immune cell function, murine models highlight the importance of suppression of activation of NF-kB, which reduces the enzymatic activity of COX-2 (15) and the synthesis of proinflammatory cytokines, mainly TNF, L-1b and IL-12 in innate immune cells (16, 17). These immunological effects of P4 are mediated by progesterone-induced blocking factor (PFIP) (18). PIBF is mainly produced by PR-positive lymphocytes during pregnancy and some malignant tumors (19). Full-length PIBF (90 kDa) participates in the regulation of the cell cycle and the invasion of trophoblast and tumor cells (20). The smaller isoforms are located in the nucleus and act as cytokines (19). The most studied immunological effects of P4 and FBIP are those related to the activity of NK lymphocytes, the decrease in their degranulation and the balance of cytokines in favor of the Th2 response (21).

Inhibitory signaling of RP has been described from studies during pregnancy, however it is not the only nuclear signaling pathway that mediates the effects of P4. One of the most important cell populations during pregnancy are uterine NK cells. (uNK) these comprise up to 70% of the leukocytes in the decidual tissues, participate in the regulation of placental development and have a decreased cytotoxic capacity (8). Uterine NK cells do not express RP, however, P4 can mediate its actions on these cells through the glucocorticoid receptor (GR) (22).

The indirect progesterone genomic pathway is mediated by membrane-bound progesterone receptors (mPRs), which interact and activate mitogen-activated protein kinases (MAPKs) to modulate gene transcription, downregulating NF-kB, which inhibits transcription. of cyclooxygenase-2 (COX-2), blocking the synthesis of arachidonic acid derivatives and attenuating the inflammatory response (23), this pathway also increases the concentration of cAMP in the MAPK pathway.

P4 inhibits the inflammatory responses of macrophages and dendritic cells (DC) through the RP or indirectly through the pattern recognition receptor and cellular synthesis of cytokines (24). PR signaling in DCs also inhibits TLR3- and TLR4-mediated IL production and the expression of costimulatory molecules (25). The data indicate that P4 mediates these effects through RP and RG. In women's T cells, mRP expression varies throughout the menstrual cycle. mPR expression is 2–5 times higher in CD8+ T cells during the luteal phase compared to the follicular phase of the cycle menstrual (26). RP signaling generally suppresses the activity of both CD4+ and CD8+ T cells, decreasing their proliferation and activation, and can trigger apoptosis (27).

In the skin, both estrogens and progesterone have effects related to the menstrual cycle and therefore to the serum concentrations of these hormones. High levels of estrogen and progesterone in the periovulatory period inhibit delayed hypersensitivity reactions, while lower levels of both hormones (perimenstruation) are associated with greater skin test reactivity, explaining exacerbations in atopic patients. These data reaffirm the role of estrogen and progesterone as inhibitors of cellular immunity (28).

## 3 Progestogen hypersensitivity, clinical presentation

Progesterone sensitization may result from IgE-mediated sensitization to endogenous progestins or with exogenous exposure to contraceptive drugs; On, special cases are patients with a history of infertility therapy or assisted reproduction, where the administration of progestogens was necessary (29). Other mechanisms, such as cross-reaction with other types of steroids such as hydrocortisone, have been proposed (30, 31).

Skin manifestations such as dermatitis or urticaria were initially reported and described as progesterone autoimmune dermatitis, although the immune-mediated mechanism was not clear. The immunological mechanisms have been described according to the previous classification of hypersensitivity, such as immediate hypersensitivity (type I), or cellular or delayed hypersensitivity (type IV), with manifestations such as skin rash, up to presentations as serious as Stevens-Johnson syndrome or presence of IgG antibodies against progesterone with the subsequent formation of immune complexes (type III hypersensitivity); However, the common characteristic is the cyclical presentation depending on the period of the menstrual cycle (32).

The clinical data suggestive of this pathology are summarized in Table 1.

**Table 1:** Clinical approach to diagnosis of PH.

	Interrogation	Physical exploration	Differential diagnosis
Clinical Record	- Cyclical pattern of symptoms (middle of the menstrual cycle) - History of assisted fertilization - Use of exogenous progestogens (contraceptives) - History of use of other types of steroids - History of atopy	- Skin symptoms (exanthema, urticaria, angioedema, eczema, purpura) - Genital inflammation - Respiratory symptoms (wheezing)	- Chronic spontaneous urticaria - Atopic dermatitis - Adverse drug reactions - Estrogens Hypersensitivity - Catamenial anaphylaxis - Breastfeeding anaphylaxis

Reference: Foer & Buchheit (33).

## 4 Diagnostic tests for progestogen hypersensitivity

### 4.1 Skin tests

Currently there is no standardization for *in vivo* or *in vitro* tests for PH diagnosis. Skin tests are performed by two techniques, prick tests and intradermal tests. Intradermal testing schemes are based on the initial concentration of the prick test, since concentrations for intradermal tests are usually ten to a thousand times lower.

The largest case series of skin testing for HP included 24 patients at Brigham and Women's Hospital; The diagnosis was made with clinical history and skin prick and intradermal tests (34). There are various clinical case reports and different proposals on how to perform this type of test in patients in whom PH is suspected, although sometimes the diagnosis is made only by consensus of experts, the skin test is performed as the initial study for evaluation (35). In our experience, skin tests can have late reactions, therefore it is necessary to maintain prolonged surveillance, and in a previous publication, the final reactivity was demonstrated by intramuscular challenge with progesterone (36).

Because PH is considered an autoimmune disease by some authors, testing such as autologous serum test, as performed in autoimmune urticaria and, has been implemented in certain cases (37); This is evidence that the immune-mediated mechanisms of the disease are not completely understood.

Recently Chamorro-Pareja et al. published a case of an 18-year-old woman who presented an adverse reaction to oral contraceptives. The reactivity occurred after 6 weeks of taking the hormones and, allergy skin tests were performed to corroborate the diagnosis. The tests were positive for progestin and estrogen. The test technique is included in Table 2 (42).

**Table 2:** Comparison of diagnostic methods with *in vivo* tests for progestogen hypersensitivity.

Author	Number of patients (*n*)	Methods	Technique	Positivity criteria	Reference
Buchheit et al.	Prospective: *n* = 24 Retrospective: *n* = 67	Prick and intradermal test	Prick: 50 mg/ml Intradermal: 0.005 mg/dl, 0.05 mg/dl, 0.5 mg/dl.	Prick: 3 mm greater than the diluent control Intradermal: 3 mm greater than the diluent control	(34, 38)
Jo et al.	*n* = 9	Prick and intradermal test	Prick: 50 mg/ml Intradermal: Not specified	Prick: progesterone wheal to histamine wheal ratio: ≥1 Intradermal: Initial wheal diameter increased by ≥3 mm after 15–20 min	(39)
Senila et al.	*n* = 1	Intradermal test	Intradermal: 5 mg/ml	Intradermal: 5 mm greater than saline control	(40)
Alonso et al.	*n* = 2	Prick, intradermal and challenge test	Prick: 50 mg/ml Intradermal: 0.05 mg/dl, 0.50 mg/dl, 5 mg/dl Intramuscular challenge: 50 mg	Prick and Intradermal: 3 mm greater than saline control Intramuscular challenge: clinical manifestations in the first hour after application	(36)
Montoro et al.	*n* = 1	Medroxyprogesterone delayed intradermal test	Intradermal: 50 mg/dl	Not specified	(41)
García et al.	*n* = 1	Prick and intradermal test with medroxyprogesterone	Prick: 20 and 2 mg/dl Intradermal: 0.002 mg/dl Autologous serum: In luteal phase	Not specified	(37)
Chamorro-Pareja et al.	*n* = 1	[Skin prick testing (SPT) and intradermal (ID)]: progesterone 50 mg/ml (SPT undiluted; ID 1: 10 000, 1: 1,000, 1: 100)	Progesterone Prick: 50 mg/ml Intradermal: 0.005 mg/ml, 0.05 mg/ml, 0.5 mg/ml Estradiol Prick: 25 mg/ml Intradermal: 0.0025 mg/ml, 0.025 mg/ml, 0.25 mg/ml	Compared with positive control (histamine), criteria for positive non specified	(42)

Only one case of cross-sensitization has been reported for one of the excipients that may contain progesterone, the product progesterone-in-oil, used for *in vitro* fertilization, was related to the presence of systemic symptoms in a 29-year-old woman, with pulmonary symptoms; In this case, no reactivity was verified by skin prick tests, intradermal or patch tests (43). This point is relevant to take into account since it is known that sesame oil may contain allergenic substances and, in this case, it would require considering testing progesterone products with different excipients (44, 45).

In Table 2 we compare different diagnostic methods using skin tests reported by different authors.

### 4.2 *In vitro* tests

Due to the clinical presentation, sometimes with immediate symptoms and other times with delayed symptoms, *in vitro* tests have been developed to determine Immunoglobulin E (IgE) and immunoglobulin G (IgG) against progesterone, given that the pathology is recognized in sometimes as allergic and other times as autoimmune.

There is no standardization to decide the positivity of the skin tests. Besides, the dilution of progesterone must be in an oily medium and, this can be irritating to the skin, thus creating false positive results. For these reasons, specific IgE direct enzyme-linked immunosorbent assay (ELISA) methods have been created to be applied in patients with suspected PH. The test was performed on patients who experienced symptoms 3–4 days before menstruation and had them resolve during or at the end of the menstrual cycle. Of the total number of individuals to whom the test was applied, 35% were atopic, presenting comorbidities such as allergic rhinitis, food allergy, drug allergy, atopic dermatitis, anaphylaxis and asthma; some of them with total IgE greater than 1,000 IU/ml. Five individuals (29.4%) had a negative result and the positive result was not related to skin testing (46).

An ELISA test was also used in the case report of a patient who was diagnosed by specific IgE for progesterone. The clinical data presented by the patient were cyclic urticaria with angioedema and, partial response to antihistamines, steroids and omalizumab. These symptoms can be classically related to IgE-mediated hypersensitivity (47). There are still no cutoff values for specific IgE for progesterone, this test cannot be considered standardized.

The identification of IgG antibodies has been a task carried out since the 1980s, first in 1982, Cheesman et al. identified 17-hydroxyprogesterone-binding immunoglobulin in the serum of a woman with cutaneous symptoms, suspected of sensitization to synthetic progestin (48).

In 1989, two cases of autoimmune dermatitis due to progesterone were reported, with pruritic erythema, edematous skin eruptions, and vesicles on the palms and soles. In addition to the skin tests performed and indirect basophil degranulation assay, an IgG-type serum factor was found; this serum factor bound to the rat corpus luteum (49). Additionally In a patient who presented erythema multiforme, immune complexes were detected after 48 h of the administration of medroxyprogesterone, reinforcing the evidence that IgG type antibodies exist for progestogens (50). These trials and results should be taken with caution since the presence of IgG, IgM, IgA and IgE antibodies has been demonstrated in allergic patients in whom the symptoms of asthma, joint pain and migraine changed in relation to the menstrual cycle or exogenous hormonal exposure (51).

As an innovative test, an ELISpot assay has been created for the detection of interferon-γ producing cells, in a report where the production of interferon-γ was demonstrated with the stimulation of PBMC in a patient with premenstrual vulvovaginal pruritus (52). To date, it is the only study that reports this innovative form of evaluation when symptoms are considered to be related to autoimmunity.

The use of tests such as basophil activation has not yet been explored and is a window of opportunity to contribute to the diagnosis of PH, in patients with symptoms that may be related to IgE-mediated hypersensitivity.

## 5 Decision to determinate the type of test to use

The decision of which test to perform should be based on the clinical symptoms and their presentation; And depending on the type of pathology, always take into account what time of the menstrual cycle the patient is at. Additionally, in the case of symptoms due to exogenous application of progestogens, the time of symptom presentation after exposure. Carrying out a challenge with the administration of endogenous progesterone requires specialized evaluation by trained personnel due to the high risk of anaphylaxis (53).

**Figure 1:**
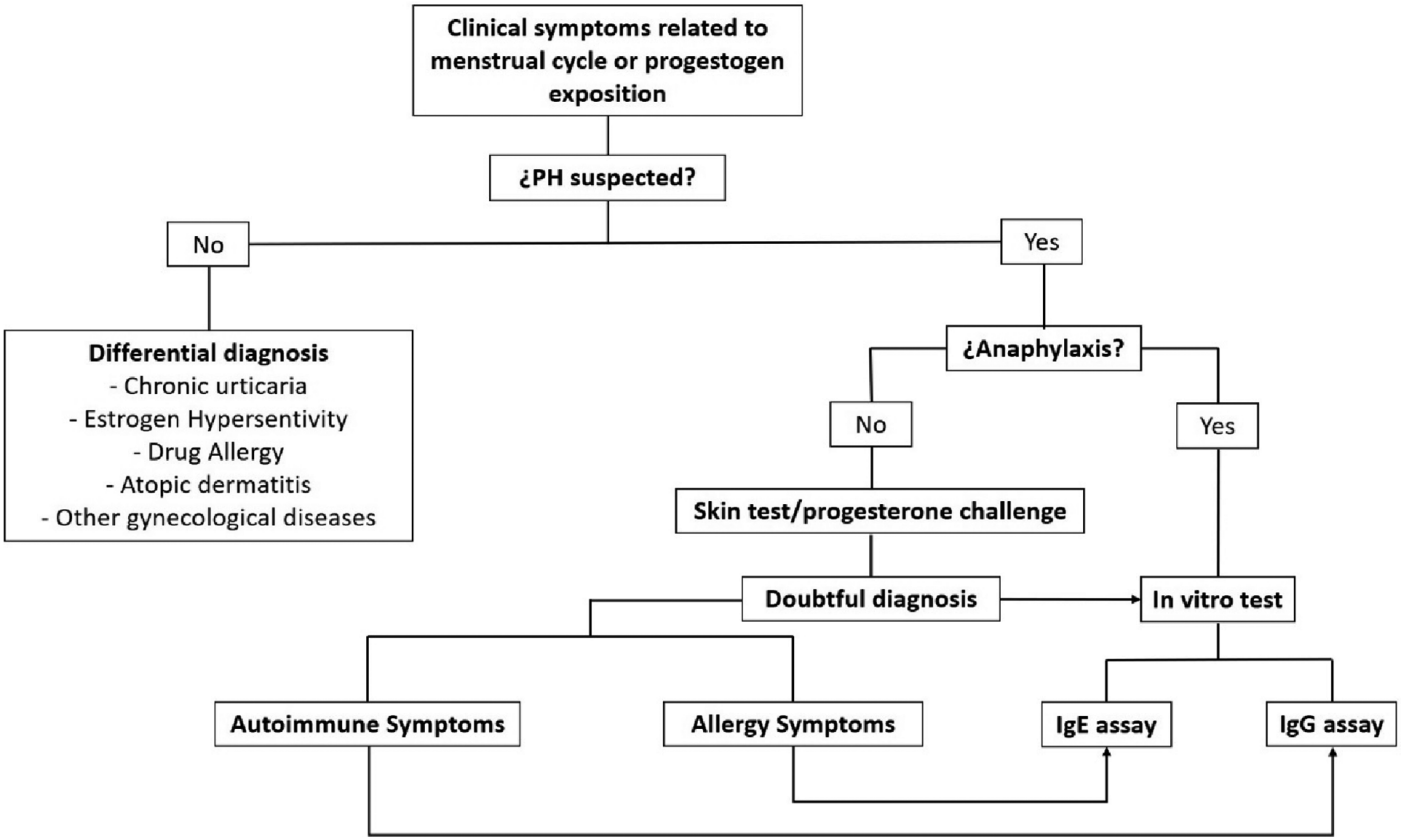
Algorithm for the use of tests to apply when PH is suspected.

Next, we propose an algorithm to determine the type of test to use when PH is suspected (Figure 1).

## 6 Conclusions

The diagnosis of PH is a diagnostic challenge; The presence of clinical symptoms should guide us by the form and time of appearance. Exogenous exposure due to therapies that require the application of progestogens may be a risk factor for developing PH.

Skin tests can guide us if they are performed with the appropriate technique and concentrations so that they are not irritating. In patients who require complementary studies, *in vitro* tests can be performed, although these are not yet commercially available.

Although the immunological mechanisms of hypersensitivity to progesterone and other hormones have not been fully described, the in-depth study of each new case published helps us learn more about the etiology of this rare pathology.
